# Crowding analysis for patients with intossication and substance abuse during the first pandemic wave of 2019 coronavirus epidemic (COVID-19) at a lombardy ED

**DOI:** 10.1192/j.eurpsy.2021.975

**Published:** 2021-08-13

**Authors:** G. Savioli, S. Pesenti, I. Ceresa, E. Oddone, M.A. Bressan

**Affiliations:** 1 Emergency Department, IRCCS Policlinico San Matteo, Pavia, Italy; 2 In Cammino Social Cooperative Of San Pellegrino Terme (bg)., La Bonne Semence social cooperative of Oltre il Colle (BG), Bergamo, Italy; 3 Department Of Public Health, University of Pavia, Pavia, Italy

**Keywords:** COVID-19 pandemic, Emergency department, crowding, intossication and substance abuse

## Abstract

**Introduction:**

The 2019 coronavirus epidemic (CoViD-19) in Italy originated in Lombardy, on February 21, 2020. Crowding has been defined as a worldwide problem as cause of reduced quality of care and patient satisfaction. It is due and identified by three orders of factors: those at the access (input); those related to the patient’s process (throughput); and those at the exit from the ED (output).

**Objectives:**

We evaluated all the population who went to ED for intossication and substance abuse. Due to the high level of care needed by these, an excessive duration of LOS (length of Stay) can be counterproductive.

**Methods:**

We evaluated all patients accessing our ED for intossication and substance abuse from February 22 to May 1, 2020 and during the same period of the previous year.

**Results:**

We enrolled 142 patients. The Crowding input factors are lower in the pandemic period: reduced attenders (41 vs 101) and reduced average waiting times (59 min vs 86 min). The Crowding throughput factors have instead worsened: LOS for both the visit rooms (810 vs 544 min) and the holding area (1205 min vs 947 min). The Crowding output factors also worsened: the percentage of access block is higher during the pandemic (10% vs 5%). The Total Access Block Time is significantly higher in the CoViD period for the holding area (1053 vs 930 min).

**Conclusions:**

The pandemic period presented a worsened crowding for these patients due to the Access Block.

